# Supplementary material for: Suicidal thoughts and behaviours in Cape Town: a cross-sectional study of prevalence, social, contextual, and clinical correlates
Source: medRxiv. 2026 Mar 12:2025.12.24.25342957. Originally published 2025 Dec 31. Preprint. [Version 2] doi: 10.64898/2025.12.24.25342957 (PMC12772667; doi:10.64898/2025.12.24.25342957)

medRxiv preprint doi: https://doi.org/10.64898/2025.12.24.25342957; this version posted March 12, 2026. The copyright holder for this preprint (which was not certified by peer review) is the author/funder, who has granted medRxiv a license to display the preprint in perpetuity.
It is made available under a CC-BY 4.0 International license .

Appendix

Table S1: Prevalence of past 30-day suicidal thoughts and behaviours, hierarchically coded, by facility

	**Facility 1**<br>N=401	**Facility 2**<br>N=201	**Facility 3**<br>N=11	**Total**<br>N=613
Past 30-day suicidal thoughts and behaviours				
None	349 (87.0%)	171 (85.1%)	7 (63.6%)	527 (86.0%)
Suicidal ideation	45 (11.2%)	15 (7.5%)	3 (27.3%)	63 (10.3%)
Suicidal plan	3 (0.7%)	5 (2.5%)	1 (9.1%)	9 (1.5%)
Suicide attempt	4 (1.0%)	10 (5.0%)	0 (0.0%)	14 (2.3%)

Data are number of participants and (percentages).

medRxiv preprint doi: https://doi.org/10.64898/2025.12.24.25342957; this version posted March 12, 2026. The copyright holder for this preprint (which was not certified by peer review) is the author/funder, who has granted medRxiv a license to display the preprint in perpetuity.
It is made available under a CC-BY 4.0 International license .

Figure S1: Co-occurrence of high perceived stress, violence exposure, and mental disorders

Panels: A) participants without suicidal ideation in the past 30 days, B) participants with suicidal ideation in the past 30 days. Areas represent approximate overlaps derived from Euler fits. Numbers indicate the number of participants in each intersection. In total, 527 participants without ideation and 86 participants with ideation were included. Mental disorder denotes the presence of any assessed current mental disorder. Violence exposure refers to lifetime exposure to violence in the family or community. High stress is defined as a Perceived Stress Score (PSS-4) ≥11. No risk factor indicates absence of all three risk factors. The intersection high stress ∩ violence exposure without mental disorder (n = 2) among participants with ideation could not be represented by the Euler fit and is therefore not shown in panel B.

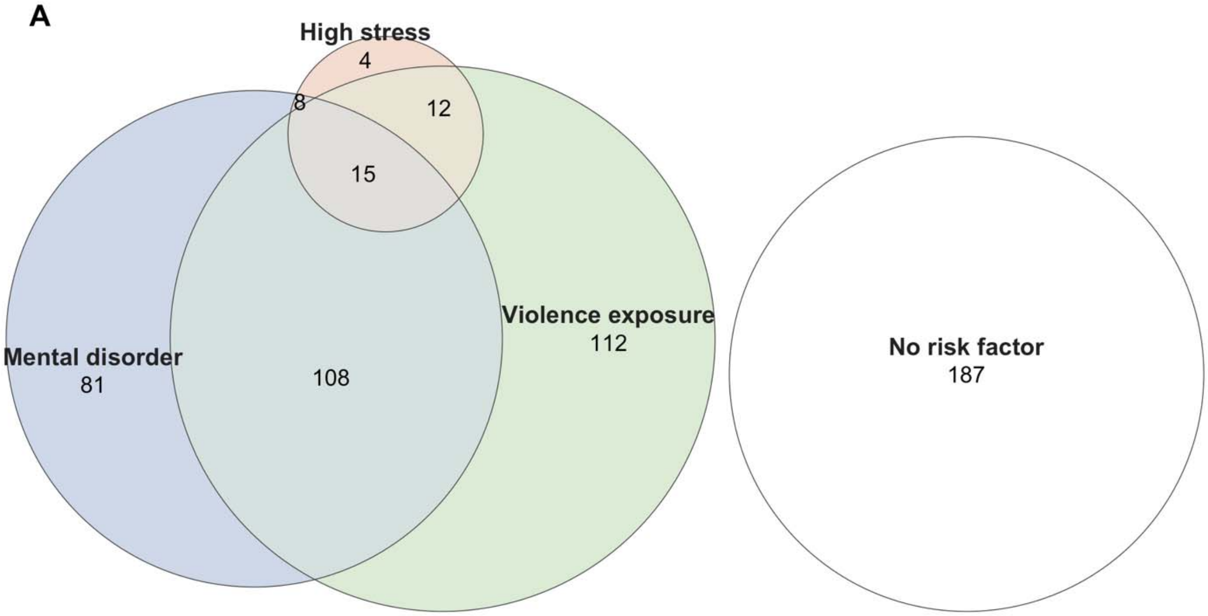

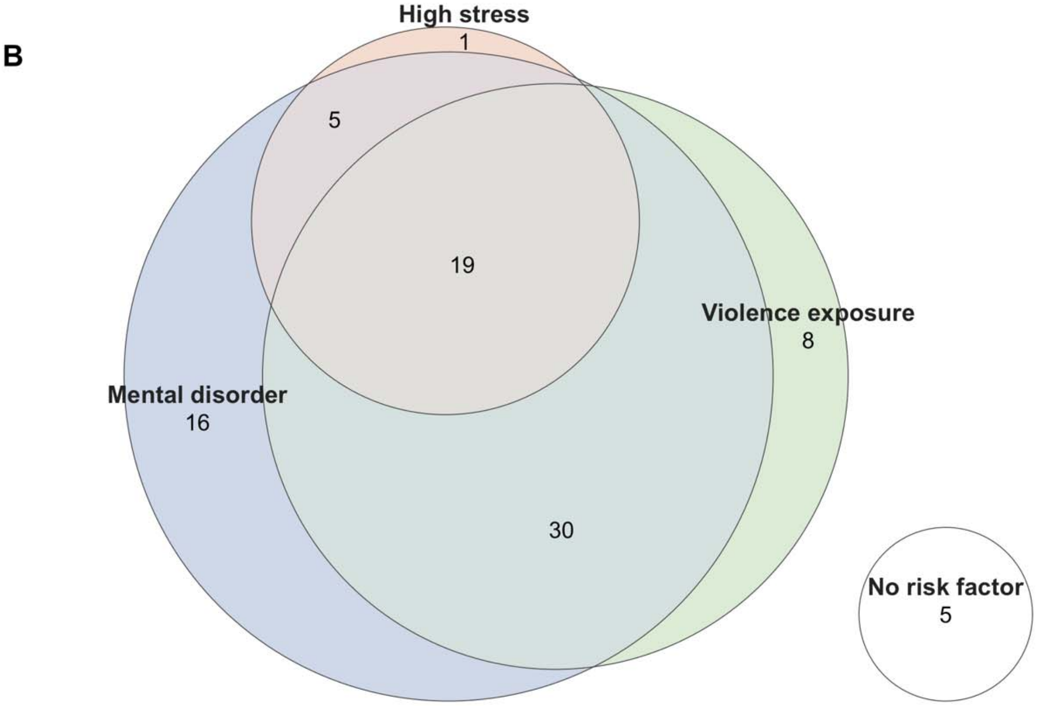

medRxiv preprint doi: https://doi.org/10.64898/2025.12.24.25342957; this version posted March 12, 2026. The copyright holder for this preprint (which was not certified by peer review) is the author/funder, who has granted medRxiv a license to display the preprint in perpetuity.
It is made available under a CC-BY 4.0 International license .

Figure S2: Associations between HIV status and past 30-day suicidal thoughts and behaviours

Adjusted odds ratios (ORs) for associations between HIV status and past 30-day suicidal ideation (black) and past 30-days suicidal thoughts and behaviours (grey). Models adjust for age group, sex, and population group. Error bars indicate 95% confidence intervals (CIs). Suicidal ideation is a binary outcome modelled with logistic regression. Suicidal thoughts and behaviours are coded hierarchically (none, ideation, plan, attempt), with participants assigned to the highest level endorsed, and modelled with ordinal logistic regression.

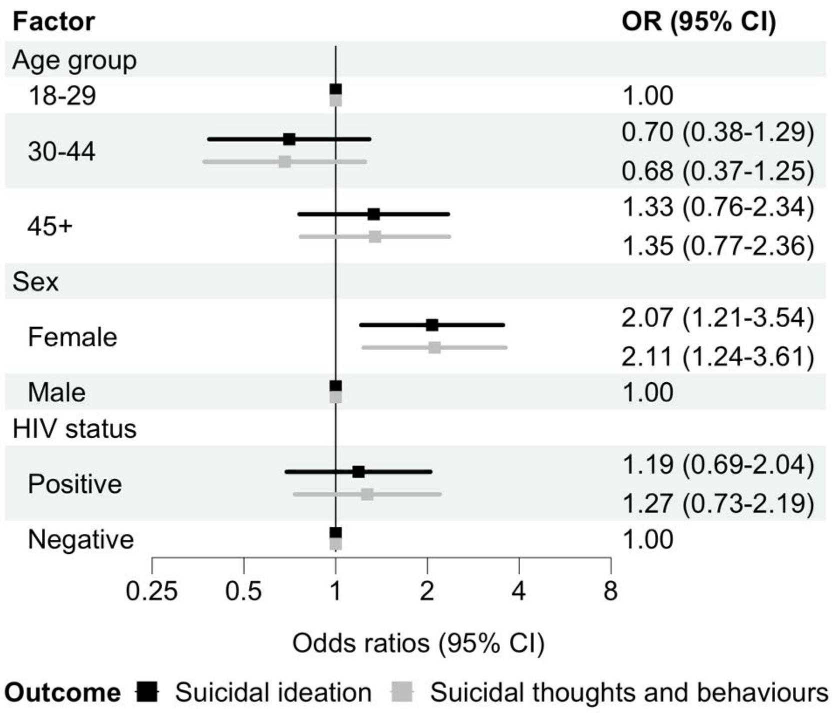

medRxiv preprint doi: https://doi.org/10.64898/2025.12.24.25342957; this version posted March 12, 2026. The copyright holder for this preprint (which was not certified by peer review) is the author/funder, who has granted medRxiv a license to display the preprint in perpetuity.
It is made available under a CC-BY 4.0 International license .

Figure S3: Sociodemographic factors and violence exposure associated with past 30-day suicidal thoughts and behaviours

Adjusted odds ratios (ORs) for associations between sociodemographic factors and violence exposure and past 30-day suicidal ideation (black) and past 30-days suicidal thoughts and behaviours (grey). Models included all sociodemographic factors shown, population group, and HIV status. Error bars indicate 95% confidence intervals (CIs). Suicidal ideation is a binary outcome modelled with logistic regression. Suicidal thoughts and behaviours are coded hierarchically (none, ideation, plan, attempt), with participants assigned to the highest level endorsed, and modelled with ordinal logistic regression.

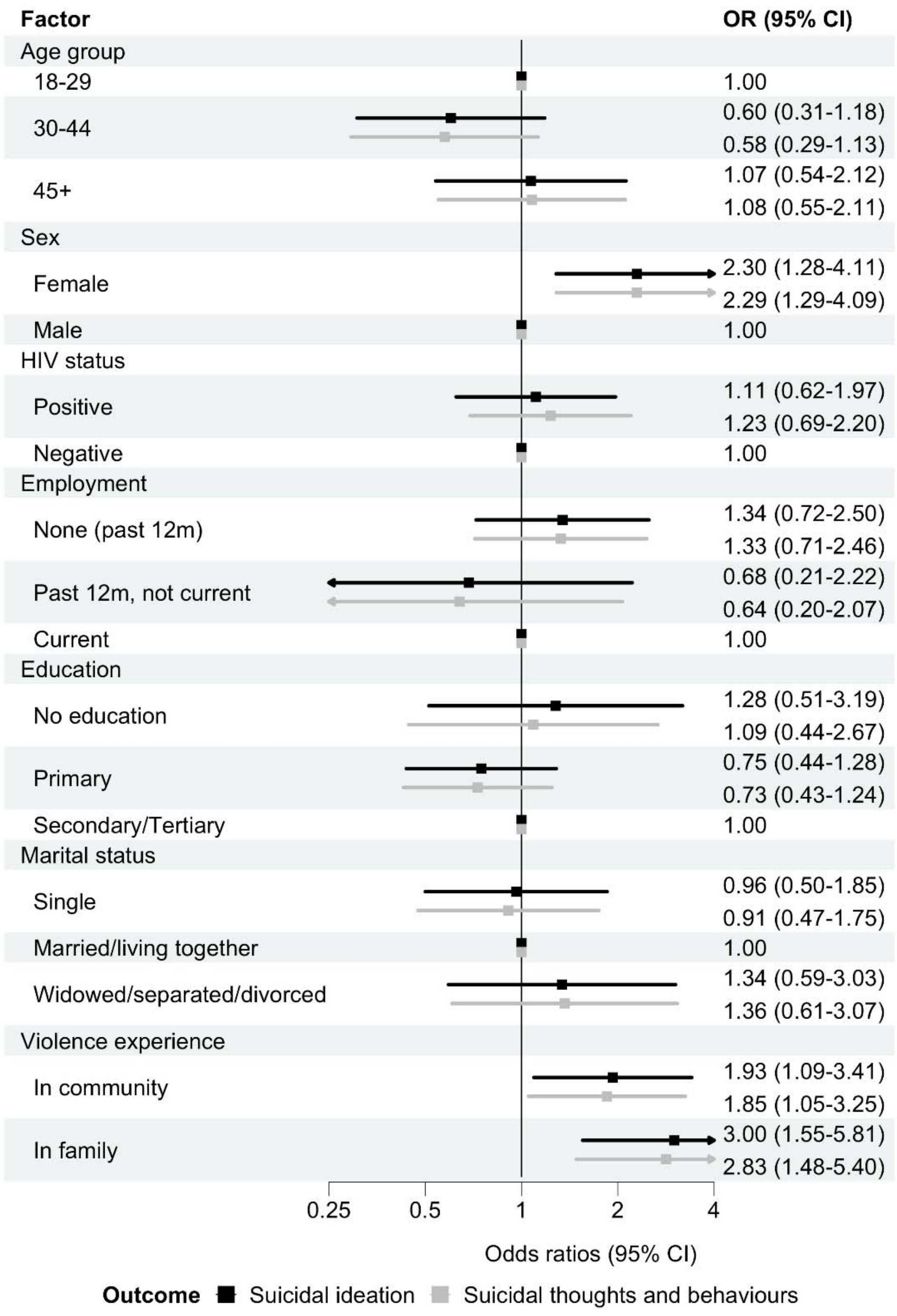

medRxiv preprint doi: https://doi.org/10.64898/2025.12.24.25342957; this version posted March 12, 2026. The copyright holder for this preprint (which was not certified by peer review) is the author/funder, who has granted medRxiv a license to display the preprint in perpetuity.
It is made available under a CC-BY 4.0 International license .

Figure S4: Associations between perceived stress and past 30-day suicidal thoughts and behaviours

Adjusted odds ratios (ORs) for associations between perceived stress and past 30-day suicidal ideation (black) and past 30-days suicidal thoughts and behaviours (grey). Models adjusted for age group, sex, population group, and HIV status. Error bars indicate 95% confidence intervals (CIs). Suicidal ideation is a binary outcome modelled with logistic regression. Suicidal thoughts and behaviours are coded hierarchically (none, ideation, plan, attempt), with participants assigned to the highest level endorsed, and modelled with ordinal logistic regression.

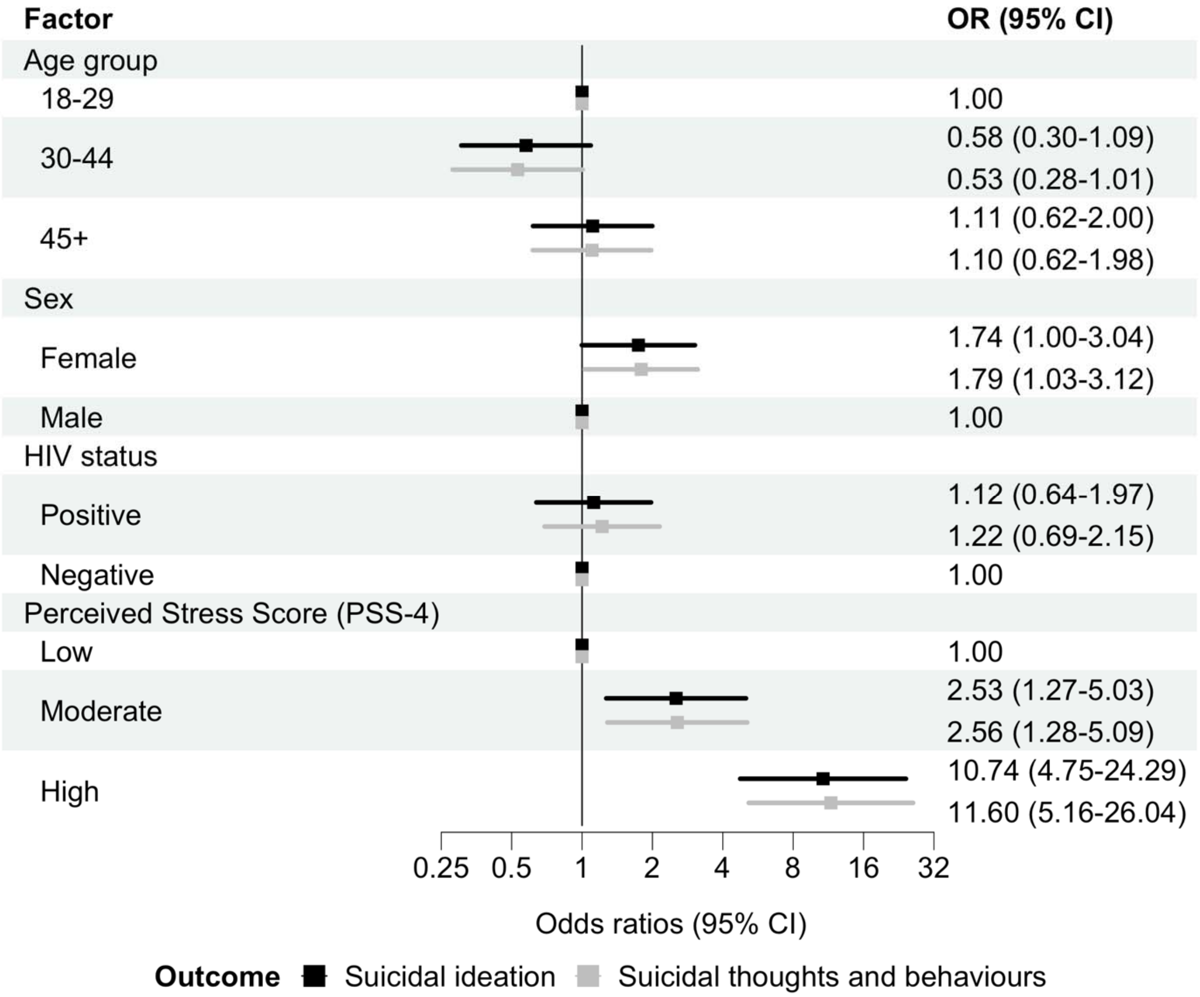

medRxiv preprint doi: https://doi.org/10.64898/2025.12.24.25342957; this version posted March 12, 2026. The copyright holder for this preprint (which was not certified by peer review) is the author/funder, who has granted medRxiv a license to display the preprint in perpetuity.
It is made available under a CC-BY 4.0 International license .

Figure S5: Associations between specific mental disorders and past 30-day suicidal thoughts and behaviours

Adjusted odds ratios (ORs) for associations between any current mental disorder and past 30-day suicidal ideation (black) and past 30-days suicidal thoughts and behaviours (grey). Models adjusted for age group, sex, population group, and HIV status. Error bars indicate 95% confidence intervals (CIs). Suicidal ideation is a binary outcome modelled with logistic regression. Suicidal thoughts and behaviours are coded hierarchically (none, ideation, plan, attempt), with participants assigned to the highest level endorsed, and modelled with ordinal logistic regression.

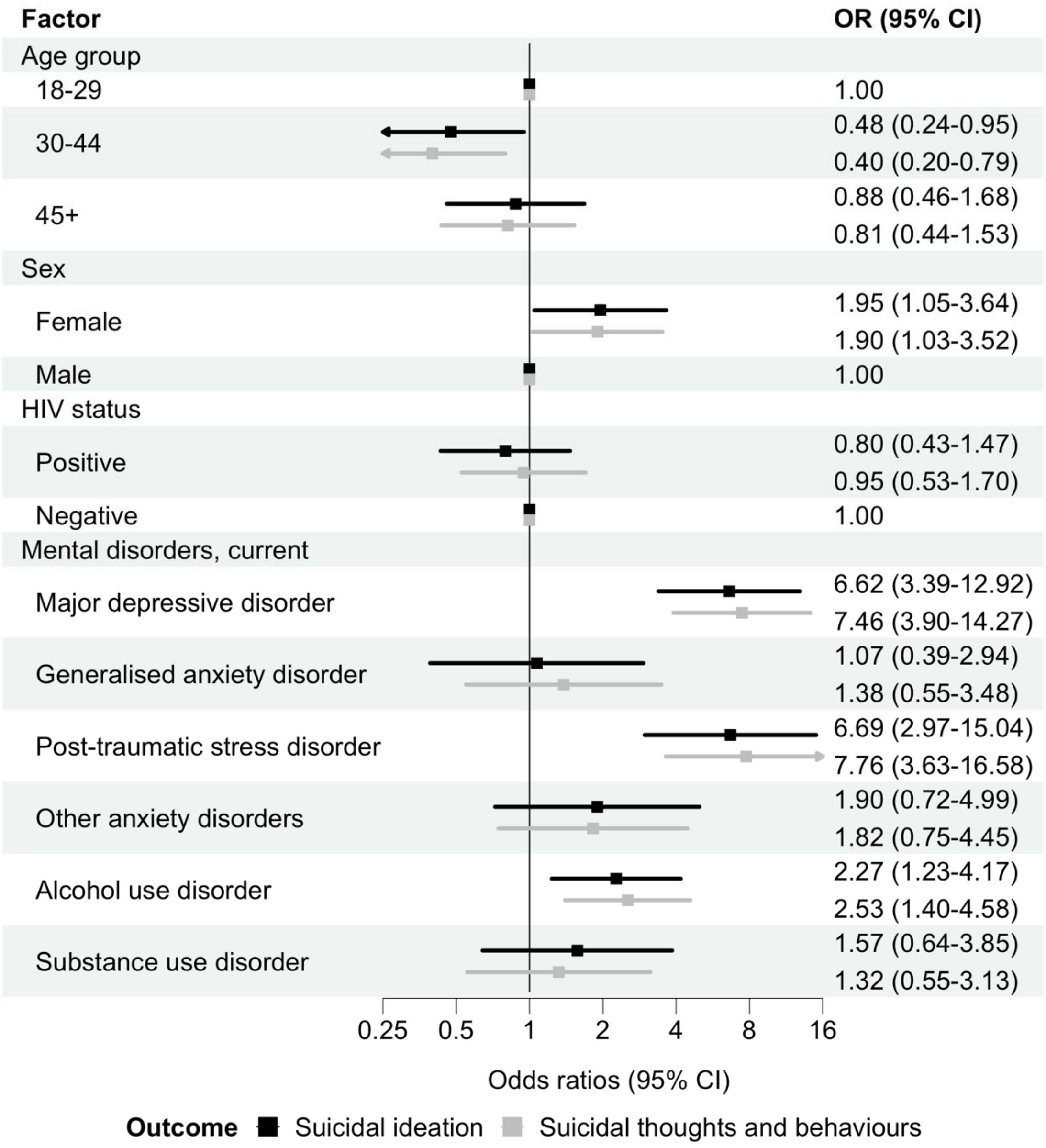

medRxiv preprint doi: https://doi.org/10.64898/2025.12.24.25342957; this version posted March 12, 2026. The copyright holder for this preprint (which was not certified by peer review) is the author/funder, who has granted medRxiv a license to display the preprint in perpetuity.
It is made available under a CC-BY 4.0 International license .

Figure S6. Average marginal effect of any current mental disorder on past 30-day suicidal ideation

Average marginal effect (AME), expressed as a percentage-point difference, for the association between any current mental disorder and past 30-day suicidal ideation. Estimates derive from an adjusted logistic regression model including age group, sex, population group, and HIV status. The AME represents the average discrete change in the predicted probability of suicidal ideation comparing participants with any current mental disorder versus none, averaged over the observed covariate distribution. Error bars indicate 95% confidence intervals. The corresponding adjusted odds ratio are presented in Figure S7.

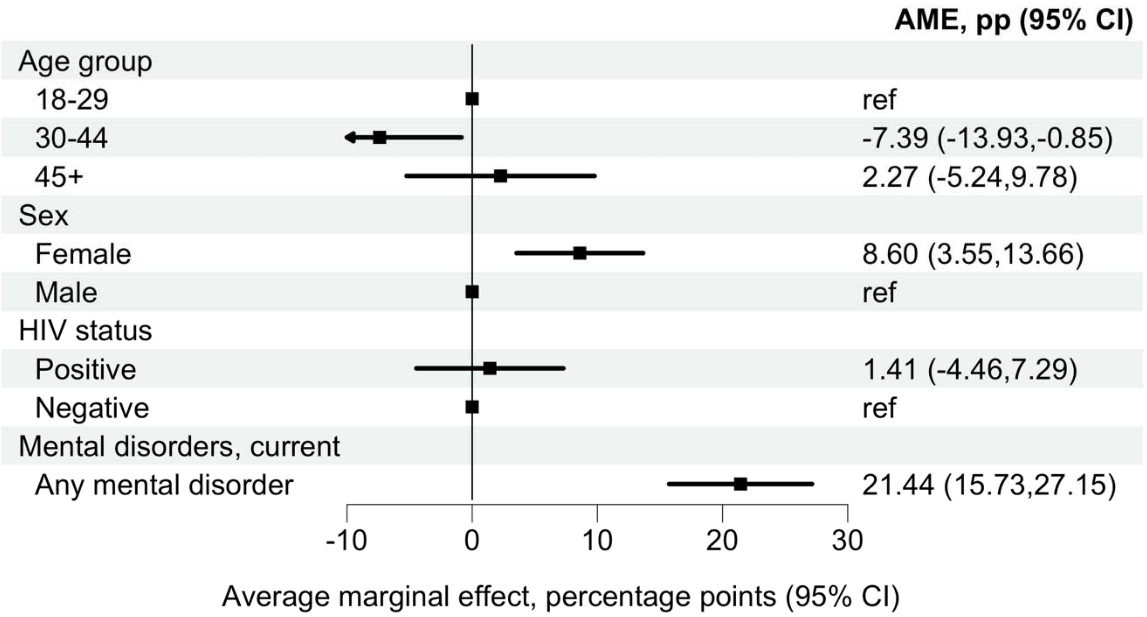

medRxiv preprint doi: https://doi.org/10.64898/2025.12.24.25342957; this version posted March 12, 2026. The copyright holder for this preprint (which was not certified by peer review) is the author/funder, who has granted medRxiv a license to display the preprint in perpetuity.
It is made available under a CC-BY 4.0 International license .

Figure S7: Associations between any mental disorder and past 30-day suicidal thoughts and behaviours

Adjusted odds ratios (ORs) for associations between any current mental disorder and past 30-day suicidal ideation (black) and past 30-days suicidal thoughts and behaviours (grey). Models adjusted for age group, sex, population group, and HIV status. Error bars indicate 95% confidence intervals (CIs). Suicidal ideation is a binary outcome modelled with logistic regression. Suicidal thoughts and behaviours are coded hierarchically (none, ideation, plan, attempt), with participants assigned to the highest level endorsed, and modelled with ordinal logistic regression.

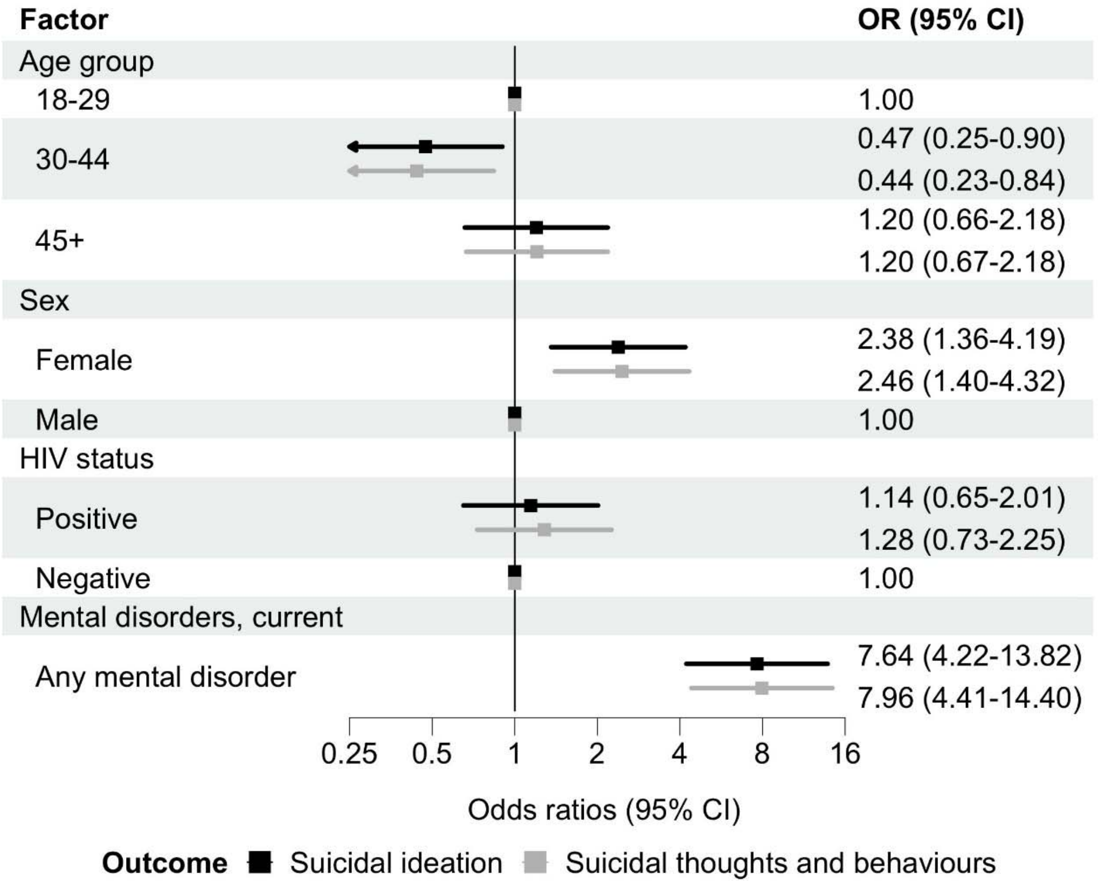

medRxiv preprint doi: https://doi.org/10.64898/2025.12.24.25342957; this version posted March 12, 2026. The copyright holder for this preprint (which was not certified by peer review) is the author/funder, who has granted medRxiv a license to display the preprint in perpetuity.
It is made available under a CC-BY 4.0 International license .

Figure S8. Average marginal effect of the number of current mental disorder on past 30-day suicidal ideation

Average marginal effects (AMEs), expressed as percentage-point differences, for the association between the number of current mental disorders and past 30-day suicidal ideation. Estimates derive from an adjusted logistic regression model including age group, sex, population group, and HIV status. AMEs represent the average discrete change in the predicted probability of suicidal ideation for each category of number of current mental disorders (1, 2, or ≥3) compared with none, averaged over the observed covariate distribution. Error bars indicate 95% confidence intervals. The corresponding adjusted odds ratio are presented in Figure S9.

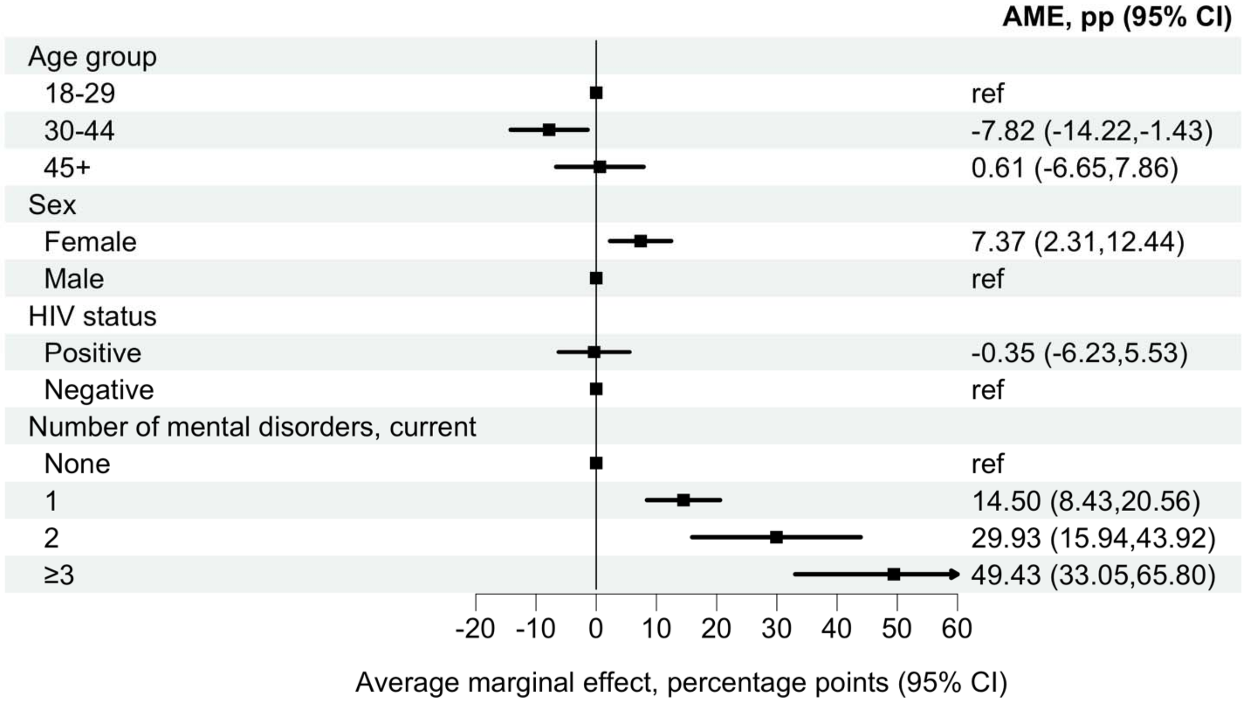

medRxiv preprint doi: https://doi.org/10.64898/2025.12.24.25342957; this version posted March 12, 2026. The copyright holder for this preprint (which was not certified by peer review) is the author/funder, who has granted medRxiv a license to display the preprint in perpetuity.
It is made available under a CC-BY 4.0 International license .

Figure S9: Associations between number of mental disorders and past 30-day suicidal thoughts and behaviours

Adjusted odds ratios (ORs) for associations between number of mental disorders and past 30-day suicidal ideation (black) and past 30-days suicidal thoughts and behaviours (grey). Models adjusted for age group, sex, population group, and HIV status. Error bars indicate 95% confidence intervals (CIs). Suicidal ideation is a binary outcome modelled with logistic regression. Suicidal thoughts and behaviours are coded hierarchically (none, ideation, plan, attempt), with participants assigned to the highest level endorsed, and modelled with ordinal logistic regression.

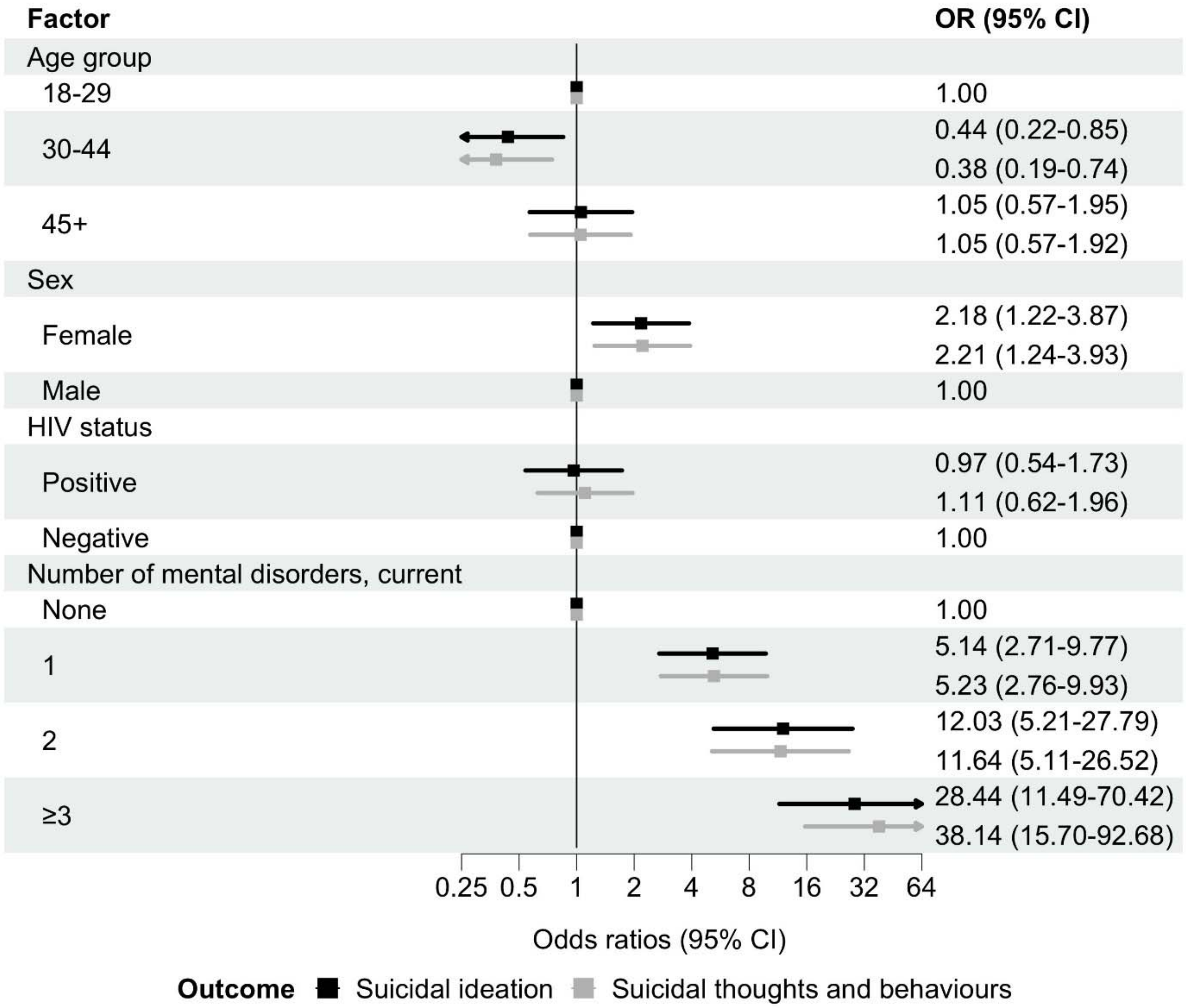

medRxiv preprint doi: https://doi.org/10.64898/2025.12.24.25342957; this version posted March 12, 2026. The copyright holder for this preprint (which was not certified by peer review) is the author/funder, who has granted medRxiv a license to display the preprint in perpetuity.
It is made available under a CC-BY 4.0 International license .

Figure S10: Associations between perceived stress and violence exposure and past 30-day suicidal thoughts and behaviours, adjusted for mental disorders

Adjusted odds ratios (ORs) for associations between perceived stress, and violence experience and past 30-day suicidal ideation (black) and past 30-days suicidal thoughts and behaviours (grey). Models adjusted for age group, sex, population group, HIV status, and the presence of any current mental disorder. Error bars indicate 95% confidence intervals (CIs). Suicidal ideation is a binary outcome modelled with logistic regression. Suicidal thoughts and behaviours are coded hierarchically (none, ideation, plan, attempt), with participants assigned to the highest level endorsed, and modelled with ordinal logistic regression.

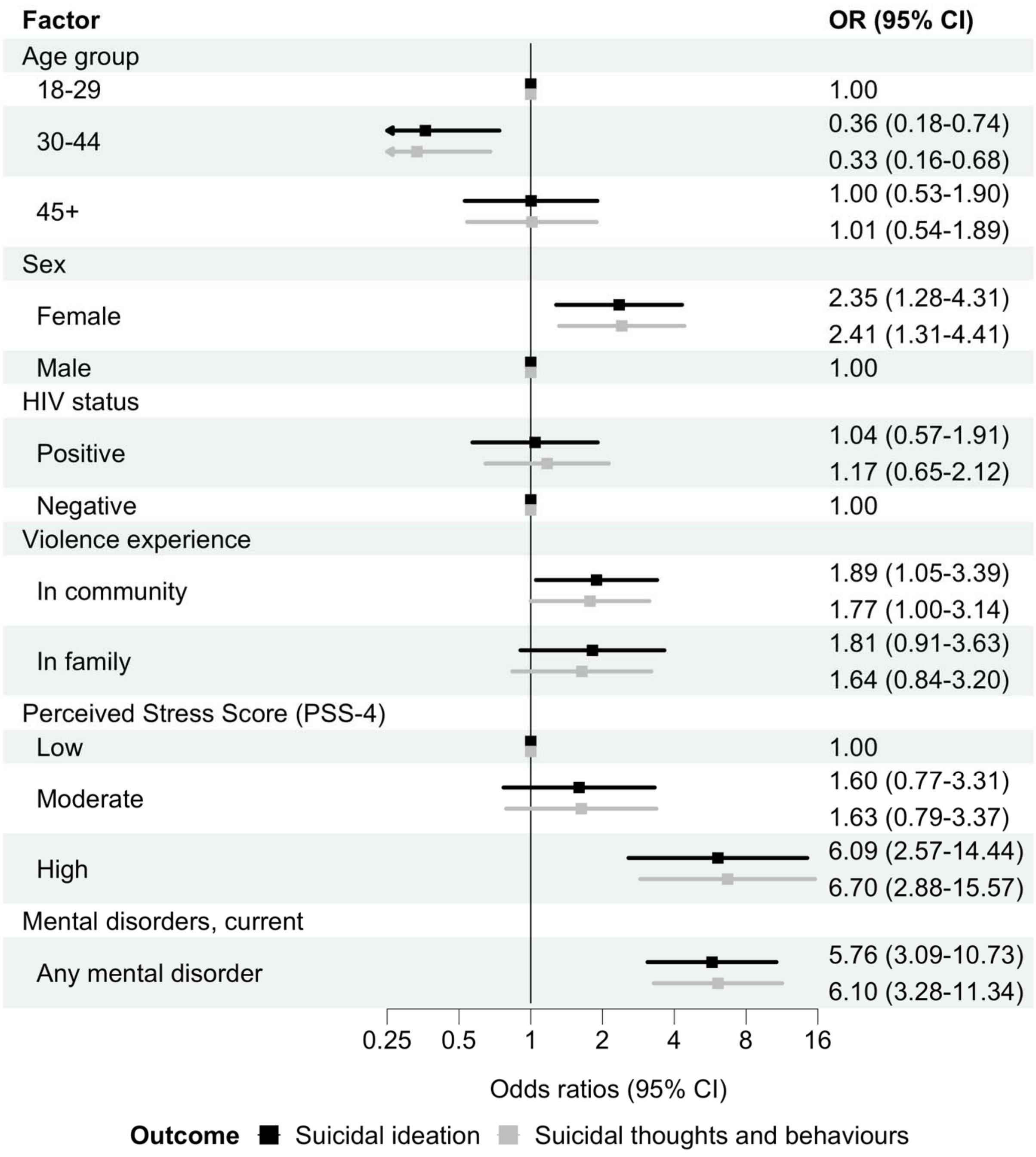

medRxiv preprint doi: https://doi.org/10.64898/2025.12.24.25342957; this version posted March 12, 2026. The copyright holder for this preprint (which was not certified by peer review) is the author/funder, who has granted medRxiv a license to display the preprint in perpetuity.
It is made available under a CC-BY 4.0 International license .

Figure S11: Unadjusted odds ratios for factors associated with past 30-day suicidal thoughts and behaviours

Unadjusted odds ratios (ORs) for factors associated with past 30-day suicidal ideation (black) and past 30-days suicidal thoughts and behaviours (grey). Error bars indicate 95% confidence intervals (CIs). Suicidal ideation is a binary outcome modelled with logistic regression. Suicidal thoughts and behaviours are coded hierarchically (none, ideation, plan, attempt), with participants assigned to the highest level endorsed, and modelled with ordinal logistic regression.

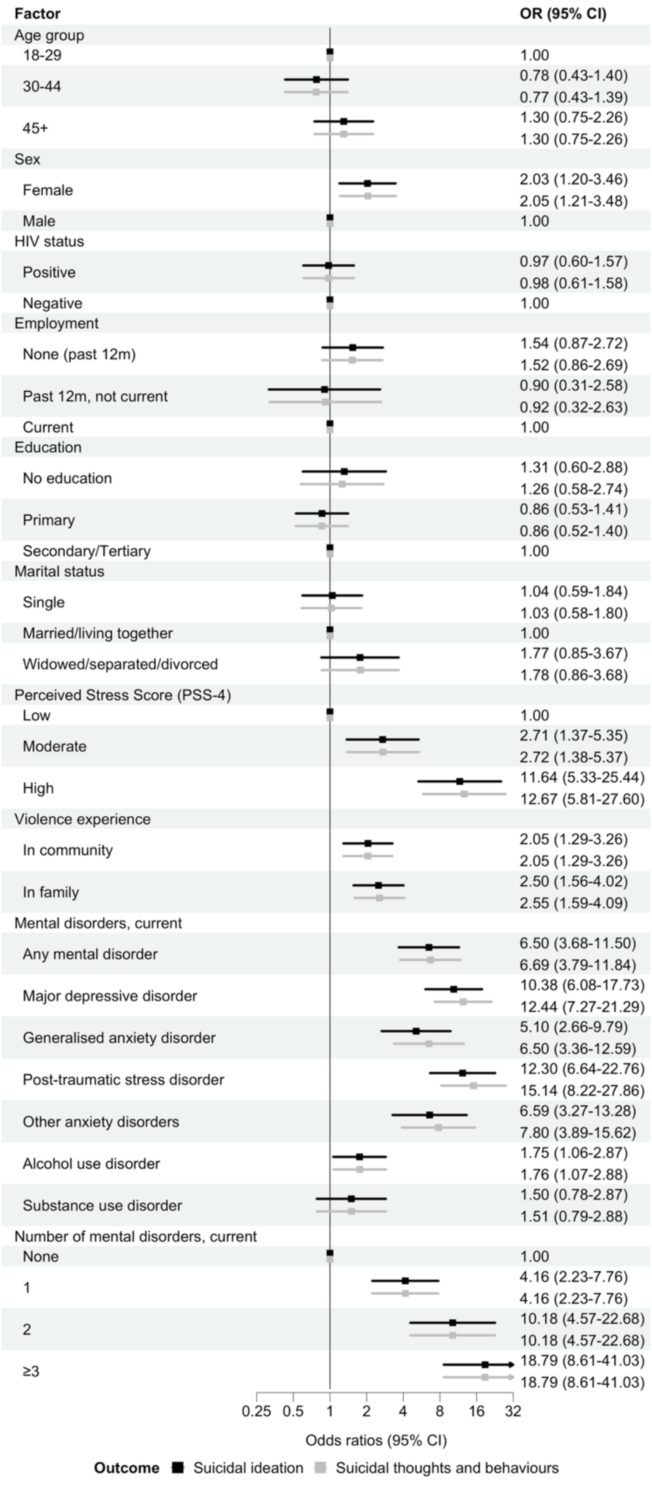

Supplement: 1 [file NIHPP2025.12.24.25342957V2-supplement-1.pdf]
