# Supplementary material for: The prevalence of suicidal thoughts and behaviours and social and clinical correlates in Cape Town: a cross-sectional study
Source: medRxiv. 2025 Dec 31:2025.12.24.25342957. Preprint. [Version 1] doi: 10.64898/2025.12.24.25342957 (PMC12772667; doi:10.64898/2025.12.24.25342957)

medRxiv preprint doi: https://doi.org/10.64898/2025.12.24.25342957; this version posted December 31, 2025. The copyright holder for this preprint (which was not certified by peer review) is the author/funder, who has granted medRxiv a license to display the preprint in perpetuity.

It is made available under a CC-BY 4.0 International license .

Appendix

Table S1: Prevalence of past 30-day suicidal thoughts and behaviours, hierarchically coded, by facility

	**Facility 1** N=401	**Facility 2** N=201	**Facility 3** N=11	**Total** N=613
Past 30-day suicidal thoughts and behaviours				
None	349 (87.0%)	171 (85.1%)	7 (63.6%)	527 (86.0%)
Suicidal ideation	45 (11.2%)	15 (7.5%)	3 (27.3%)	63 (10.3%)
Suicidal plan	3 (0.7%)	5 (2.5%)	1 (9.1%)	9 (1.5%)
Suicide attempt	4 (1.0%)	10 (5.0%)	0 (0.0%)	14 (2.3%)

Data are number of participants and (percentages).

Figure S1: Co-occurrence of high perceived stress, violence exposure, and mental disorders

medRxiv preprint doi: https://doi.org/10.64898/2025.12.24.25342957; this version posted December 31, 2025. The copyright holder for this preprint (which was not certified by peer review) is the author/funder, who has granted medRxiv a license to display the preprint in perpetuity.

It is made available under a CC-BY 4.0 International license .

Panels: A) participants without suicidal ideation in the past 30 days, B) participants with suicidal ideation in the past 30 days. Areas represent approximate overlaps derived from Euler fits. Numbers indicate the number of participants in each intersection. In total, 527 participants without ideation and 86 participants with ideation were included. Mental disorder denotes the presence of any assessed current mental disorder. Violence exposure refers to lifetime exposure to violence in the family or community. High stress is defined as a Perceived Stress Score (PSS-4) ≥11. No risk factor indicates absence of all three risk factors. The intersection high stress ∩ violence exposure without mental disorder (n = 4) among participants with ideation could not be represented by the Euler fit and is therefore not shown in panel B.

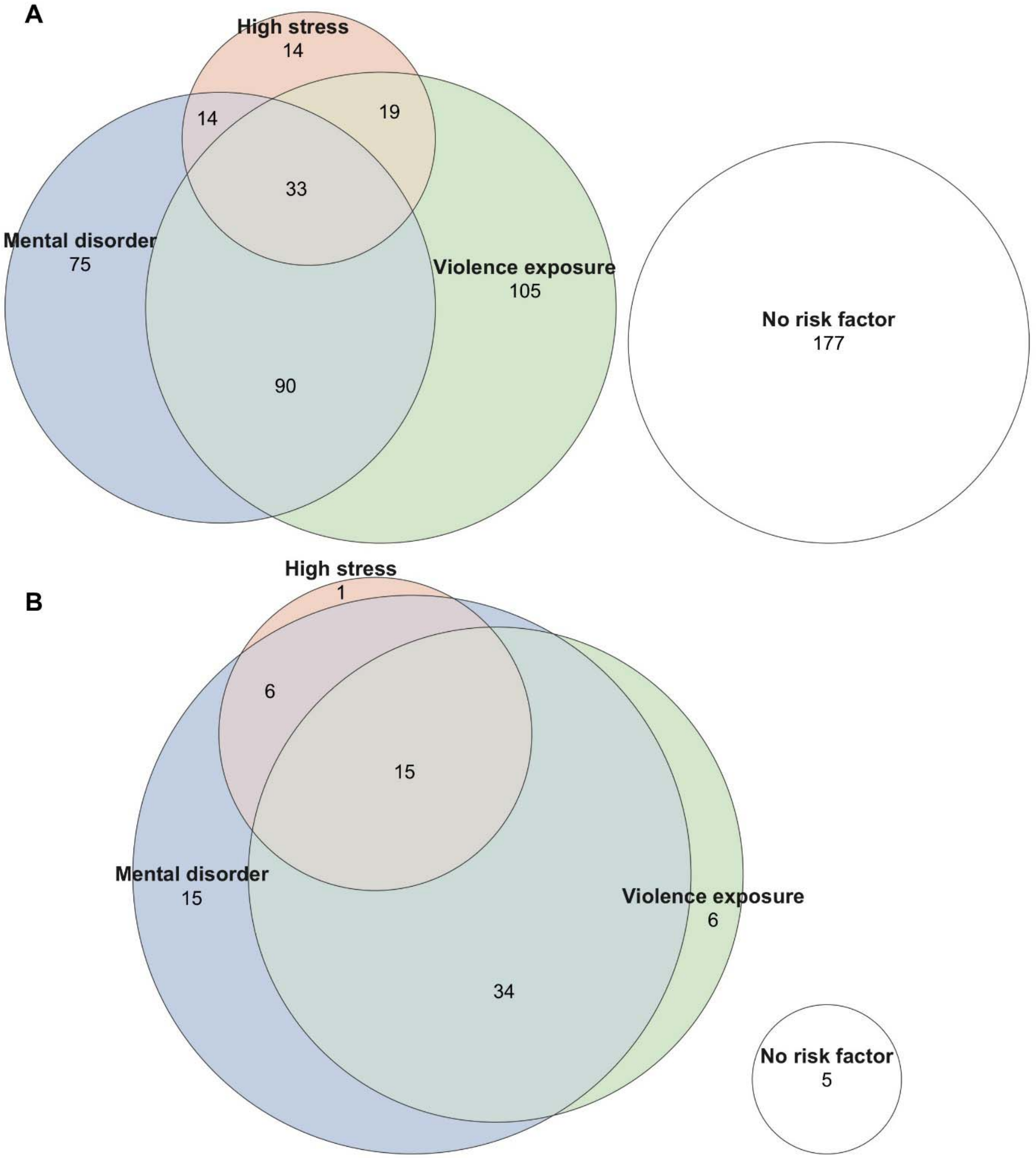

medRxiv preprint doi: https://doi.org/10.64898/2025.12.24.25342957; this version posted December 31, 2025. The copyright holder for this preprint (which was not certified by peer review) is the author/funder, who has granted medRxiv a license to display the preprint in perpetuity.

It is made available under a CC-BY 4.0 International license .

Figure S2: Associations between any mental disorder and past 30-day suicidal thoughts and behaviours

Adjusted odds ratios (ORs) for associations between any current mental disorder and past 30-day suicidal ideation (black) and past 30-days suicidal thoughts and behaviours (grey). Models adjusted for age group, sex, population group, and HIV status. Error bars indicate 95% confidence intervals (CIs). Suicidal ideation is a binary outcome modelled with logistic regression. Suicidal thoughts and behaviours are coded hierarchically (none, ideation, plan, attempt), with participants assigned to the highest level endorsed, and modelled with ordinal logistic regression.

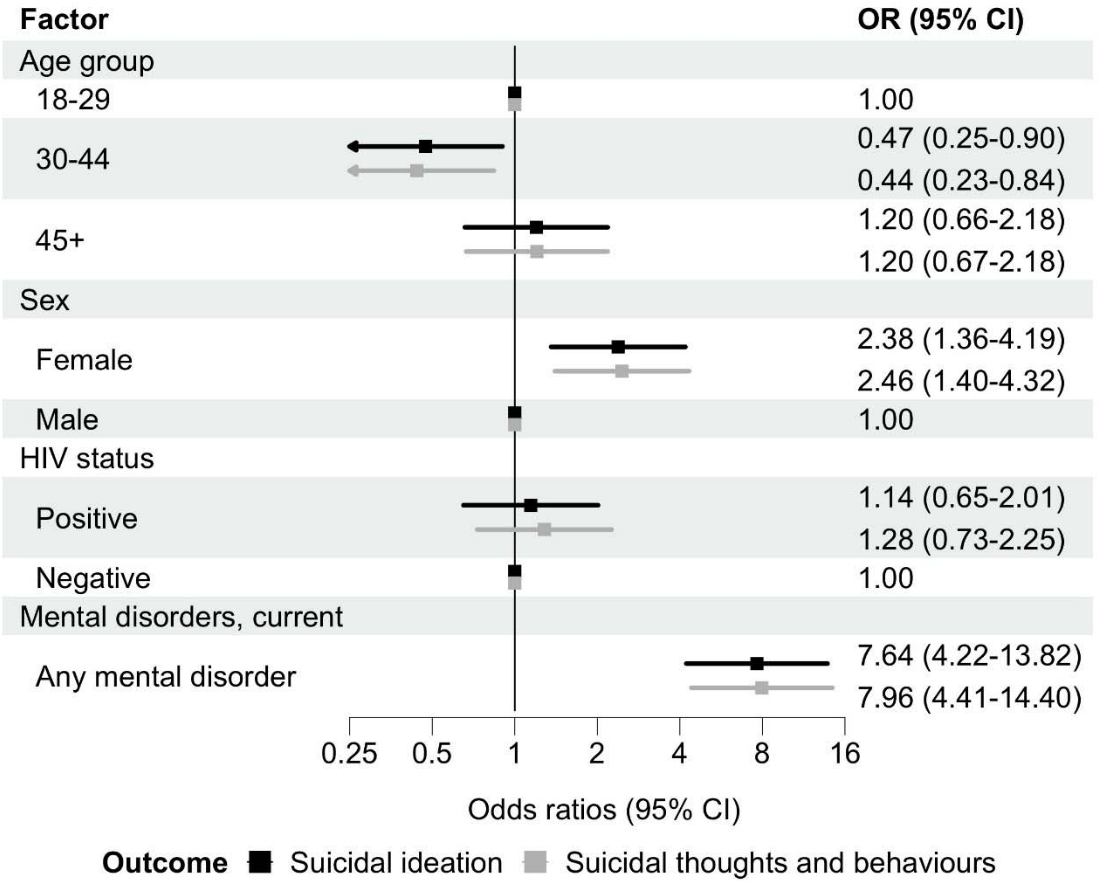

medRxiv preprint doi: https://doi.org/10.64898/2025.12.24.25342957; this version posted December 31, 2025. The copyright holder for this preprint (which was not certified by peer review) is the author/funder, who has granted medRxiv a license to display the preprint in perpetuity.

It is made available under a CC-BY 4.0 International license .

Figure S3: Associations between number of mental disorders and past 30-day suicidal thoughts and behaviours

Adjusted odds ratios (ORs) for associations between number of mental disorders and past 30-day suicidal ideation (black) and past 30-days suicidal thoughts and behaviours (grey). Models adjusted for age group, sex, population group, and HIV status. Error bars indicate 95% confidence intervals (CIs). Suicidal ideation is a binary outcome modelled with logistic regression. Suicidal thoughts and behaviours are coded hierarchically (none, ideation, plan, attempt), with participants assigned to the highest level endorsed, and modelled with ordinal logistic regression.

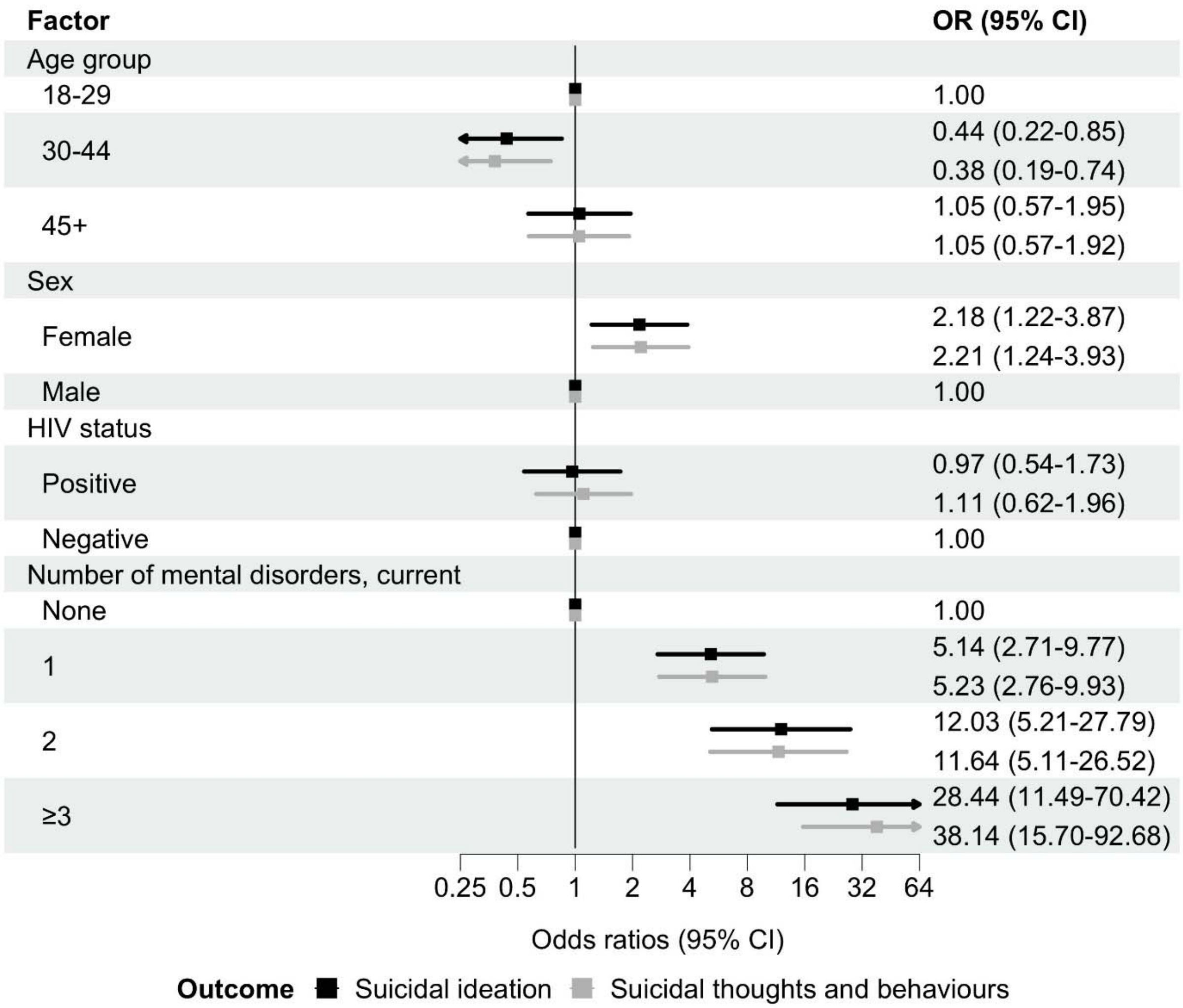

medRxiv preprint doi: https://doi.org/10.64898/2025.12.24.25342957; this version posted December 31, 2025. The copyright holder for this preprint (which was not certified by peer review) is the author/funder, who has granted medRxiv a license to display the preprint in perpetuity.

It is made available under a CC-BY 4.0 International license .

Figure S4: Unadjusted odds ratios for factors associated with past 30-day suicidal thoughts and behaviours

Unadjusted odds ratios (ORs) for factors associated with past 30-day suicidal ideation (black) and past 30-days suicidal thoughts and behaviours (grey). Error bars indicate 95% confidence intervals (CIs). Suicidal ideation is a binary outcome modelled with logistic regression. Suicidal thoughts and behaviours are coded hierarchically (none, ideation, plan, attempt), with participants assigned to the highest level endorsed, and modelled with ordinal logistic regression.

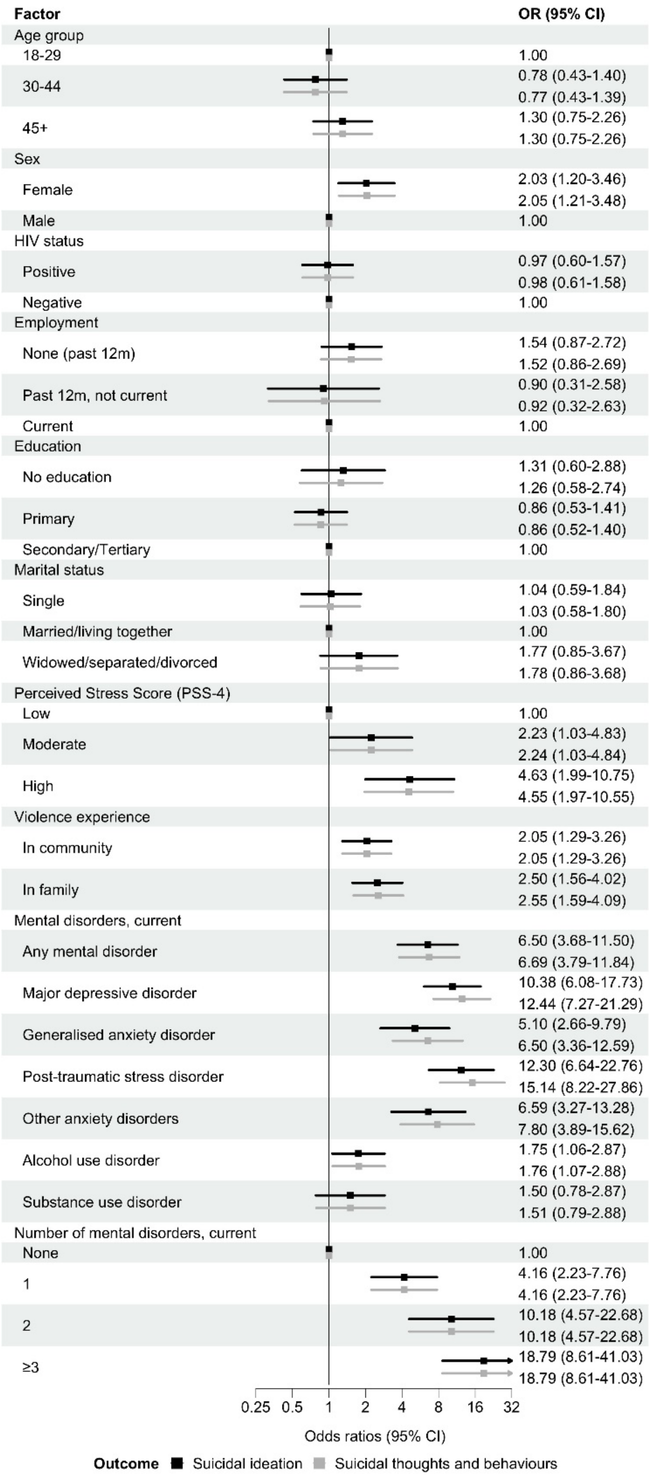

Supplement: Supplementary file 1 [file NIHPP2025.12.24.25342957V1-supplement-1.pdf]
